# The contribution of nursing doctoral schools to the development of evidence 10 years after their establishment in Italy: An exploratory descriptive survey of former and current doctoral students’ publications

**DOI:** 10.1002/nop2.262

**Published:** 2019-03-21

**Authors:** Loredana Sasso, Roger Watson, Michela Barisone, Ramona Pellegrini, Fiona Timmins, Giuseppe Aleo, Valentina Bressan, Monica Bianchi, Lucia Cadorin, Nicoletta Dasso, Dario Valcarenghi, Gianluca Catania, Milko Zanini, Annamaria Bagnasco

**Affiliations:** ^1^ Department of Health Sciences University of Genoa Genoa Italy; ^2^ Faculty of Health and Social Care University of Hull Hull UK; ^3^ School of Nursing and Midwifery Trinity College Dublin Dublin Ireland; ^4^ Department of Otolaryngology/Head & Neck Surgery University Hospital Santa Maria della Misericordia, Azienda Sanitaria Universitaria Integrata di Udine Udine Italy; ^5^ University of Applied Sciences and Arts of Southern Switzerland Department of Business Economics, Health and Social Care Stabile Piazzetta Via Violino Manno Switzerland; ^6^ CRO Aviano National Cancer Institute Aviano Italy; ^7^ Oncology Institute of Southern Switzerland San Giovanni Hospital Bellinzona Switzerland; ^8^ School of Nursing University of Pennsylvania Philadelphia Pennsylvania

**Keywords:** competencies, decision‐making, doctoral nursing education, nursing research, policy, research knowledge, survey

## Abstract

**Aim:**

To analyse through an exploratory descriptive survey how former and current doctoral students’ publications have contributed to the development of evidence between the establishment of the doctoral schools of nursing in 2006–2015.

**Design:**

An exploratory descriptive survey.

**Methods:**

We analysed the papers published in peer‐reviewed journals by the four Italian PhD Schools of Nursing between 2006–2015. Additional missing information was retrieved from Web of Science.

**Results:**

We identified 478 scientific papers. The papers increased from 12 in 2006–110 in 2015. Most are published in 29 journals, of which 15 had an impact factor ranging between 0.236–3.755. These results show the increasingly significant contribution of nursing doctoral programmes to the production of evidence, which can be used to improve the quality of nursing and inform health policies. Nursing doctoral schools deserve a greater recognition, especially by Italian funding agencies and political institutions.

## 1 INTRODUCTION

In the last 20 years, the nursing profession has evolved so much that it has radically changed the way of being a nurse, shifting the focus of professional practice from exclusively clinical areas to academia and research (Palese et al., 2014). In the past, nurses preferred and were encouraged to gain clinical experience by working before pursuing graduate education. This clinical emphasis also resulted in the requirement to earn a master's degree in a specialty and obtain more clinical experience before pursuing PhD education (Ellembecker & Kazmi, 2014).

The new and challenging prospects of nursing are the result of research and innovation conducted by senior lecturers and researchers, who are now playing strategic education roles to determine the lines of future development in the social and healthcare fields in synergy with the new generations of professionals (Cheraghi, Jasper, & Vaismoradi, 2014; Holland, 2015).

Nurses who access the highest levels of academic education, such as doctoral students, are called on by the international scientific community to take an active part in the production of new knowledge and to promote the culture of evidence‐based practice through project development competencies, critical analysis and innovative ideas that adequately respond to the emerging needs of the health system (Begley, McCarron, Huntley‐Moore, Condell, & Higgins, 2014). Supporting and fostering the production of scientific publications means creating innovation, quality, more opportunities for international cooperation, more interdisciplinary collaboration and making a discipline more appealing for members inside and outside the same profession. This involves the development of a culture of research, which in the field of nursing requires the attainment of a substantial number of nurses with a doctoral degree, financial support and a strong local and national leadership (Begley et al., 2014).

### 1.1 Background

The American Association of Colleges of Nursing (AACN, 2016) and other institutions across the world have increasingly focused on the development of doctoral schools of nursing and launched debates on the development of academic programmes to prepare future generations of nurses who are experts in research (AACN, 2016). In the USA, for instance, the number of nurses with doctoral education has doubled since 1960, when the first nursing doctoral courses were established, reaching their maximum development in the 1980s. Initially, nursing research was part of the National Institutes of Health, but then it established its own National Institute for Nursing Research (NINR) (Ketefian, Davidson, Daly, Chang, & Srisuphan, 2005). In fact, the NINR in the United States has funded nursing research through educational programmes to support research and researchers to study phenomena associated with health care directly provided to the person, through the same funding systems of the National Institutes of Health (AACN, 2016). Therefore, in the United States, thanks to the scientific rigour and methodological soundness of the research conducted by the doctoral schools of nursing, these now play a key role for the funding system of the US health policies.

Also, in Europe, the nursing profession is increasing its level of education, professional competencies and its role in research (Bressan et al., 2016). Just like any other health profession, the development of the culture of research requires the attainment of PhDs, funding and support at a national level and leadership and vision at the academic level (Begley et al., 2014). Indeed, several studies have shown how appropriate preparation and acquisition of knowledge in the field of research through doctoral programmes can facilitate the development of clinical competencies and bring nursing practice to a higher level (Begley et al., 2014; Bressan et al., 2016). Through research, the creation of databases and the validation of tools, doctoral programmes offer clinical nurses the opportunity to increase their knowledge and, consequently, provide them with the potential to ensure higher standards of patient safety (Cheraghi et al., 2014).

With regard to the Italian context, a “doctorate” is defined as the level of education that “provides the necessary competencies to conduct high‐quality research for public and private bodies, as well as to practice as independent professionals, contributing to the accomplishment of the European Higher Education Area and to the European Research Area” (Decree of the Italian Ministry of Education, University, & Research, 2013).

Access to a doctoral programme is ruled by admission criteria and tests designed to select highly prepared candidates, who can innovate. These characteristics imply the willingness and the ability to complete a complex and difficult programme, which will enable students to achieve the highest level of education available today. After completing the PhD programme, students will have the competencies to contribute to national and international scientific projects and be competitive in the labour market by having access to high‐level job positions in all the available fields of a profession (Stiwne & Alves, 2010). This reinforces the roles played by Italian nurse academics, who are ensuring that nurses develop increasingly higher research competencies (Bagnasco, Ghirotto, & Sasso, 2014).

In the past, publications in the field of nursing research were very few, and in many cases, these studies were not conducted by nurses, but by other health professionals. Instead, recently the number of studies and scientific papers published by Italian nurses has steadily increased (Marucci et al., 2013). Advanced competencies in the field of research are needed to publish scientific papers that show the international scientific community the value of the studies conducted, share new scientific evidence and ultimately enable to highlight the importance of PhD schools through their publications, which have become a measure of the quality of academic excellence (Langer, 2017).

Since, the doctoral nursing programmes were launched in Italy for the first time in 2006, after 10 years we felt that it would be important to analyse and highlight how nursing research has developed through the work of all of the four PhD Schools of Nursing in Italy. This would provide the scientific evidence necessary to facilitate, for instance, discussions with healthcare policymakers and help to bring nursing practice to a higher level. Therefore, the research question of this study was: “How have nursing doctoral schools contributed to the development of nursing research in the last 10 years?”

## 2 THE STUDY

### 2.1 Design

This is an exploratory descriptive survey conducted on a national level that analysed publications produced from 2006–March 2016 by current and former doctoral students of the four PhD Schools of Nursing across Italy.

### 2.2 Sample

The sample of this study was a convenience sample of the publications of current and former doctoral students from all four PhD Schools of Nursing in Italy.

### 2.3 Data collection

We involved all four PhD Schools of Nursing present in Italy by email and invited current and former PhD nursing students to complete a questionnaire consisting of 25 items, which was specifically designed by our research team for this study.

Participants were asked to provide data regarding the year they started their doctoral programme (i.e. cycle), the date they completed it, specifying if they had finished on time or if they were overdue, their publications before, during and after their doctoral studies and job positions covered before and after completing their PhD programme.

For each author, a table was built to collect data on the respective publications, such as authorship, if the journals were peer‐reviewed, number of citations, impact factors and number of publications on national and international journals with an impact factor. We preliminarily contacted the coordinators of all the PhD Schools of Nursing by phone asking them to provide the lists of all their current and former PhD students and their mailing lists. Then, we contacted the students asking them to provide within 10 days a copy of their curriculum vitae containing a detailed list of all their publications. We sent two invitations to obtain the highest possible number of participants. Finally, we checked all the publication lists sent by the PhD students with those available in Web of Science to add extra information regarding the journal impact factor, year of publication and authorship.

### 2.4 Data analysis

Through the online survey, all the data were automatically collected into a table created by the online survey software. Afterwards, the data were entered into an Excel file, which included also the additional data we collected from Web of Science. The database contained some general information provided by each respondent from the four PhD Schools (starting date, number of publications, position covered before and after the doctoral programme, their educational background, etc.) and the data regarding all the publications for each of the four doctoral schools of nursing. The data analyses were conducted in three macro areas: number of publications in scientific journals for each PhD School, publication topics and publication trends between 2006–2015.

### 2.5 Statistical analysis

Statistical analysis was conducted using Microsoft Excel 2010 and the Statistical Package for Social Science (SPSS for Windows, IBM, New York) version 21.0. To study the statistical distribution of the areas under investigation, we adopted the descriptive statistics methodology (mean and standard deviation). Conference proceedings and duplicates were excluded. All the remaining publications were grouped together according to the PhD School they belonged to and then analysed.

### 2.6 Ethical approval

Since this study was conducted on publicly available papers and in agreement with Italian national legislation on research studies, no Research Ethics Committee approval was required by the local ethics committee. However, permission to conduct this study was obtained by the Internal Review Board of our university. An email was sent to invite all potential participants, informing them about the aims of the research and in what way the collected data would have been handled and processed. Participation in the study was exclusively voluntary, and written consent was obtained through acceptance to participate and the completion of the questionnaire. All the data collected for this study were coded to ensure anonymity.

## 3 RESULTS

### 3.1 Sample

The total number of respondents was 79. Of these, 48 were from the PhD School of Rome, 19 from Genoa, seven from Florence and five from L'Aquila. The characteristics of the respondents are shown in Table 1.

**Table 1:** Characteristics of the respondents from each PhD School of Nursing

PHD school	Genoa	L'Aquila	Florence	Rome
Respondents	19	5	7	48
Age (average)	43.7	41.4	36.3	41.8
Beginning of the PhD				
From 2006–2009	6	1	0	4
From 2010–2012	9	3	0	24
From 2013–2015	4	1	7	20
Obtaining the degree				
2010	1	1	0	1
2013	3	0	0	1
2014	2	1	0	6
2015	3	1	0	4
2016	4	0	0	0
Degree obtained before PhD				
Master of Nursing Science	19	5	7	48
Master degree in nursing management	5	2	4	11
Others master degrees	2	1	1	5
Professional role before the PhD				
Nurse/midwife	6	2	3	30
Nurse/midwife ward manager	8	2	3	10
Academic tutor	3	0	1	6
Researcher	1	0	0	1
Student	0	1	0	1

### 3.2 Publications between 2006–2015

We collected 701 scientific articles. Of these, 699 (99.71%) were already published and 2 (0.29%) were in submission or in press. After removing the duplicates, we remained with 478 papers, of which 476 (99.58%) were already published and 2 (0.42%) were in press.

### 3.3 Distribution of the scientific publications

**Figure 1:**
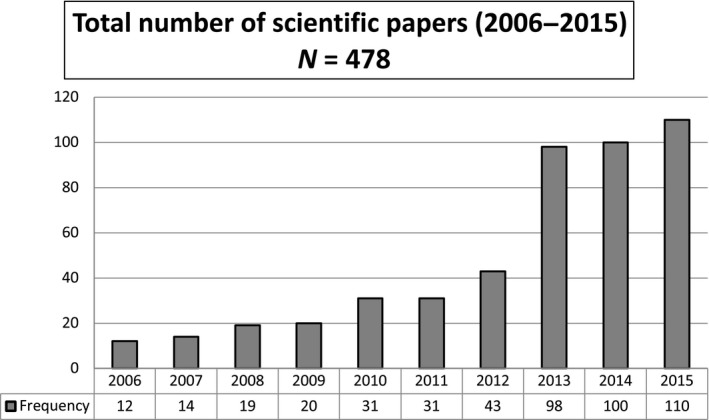
Total number of scientific papers (2006–2015)

Of all the scientific articles, 226 (47.59%) were published in Italian national journals and 250 (52.41%) in international journals. The scientific papers were published in a total of 170 journals, and of these, 83 (48.82%) had an impact factor. The total number of papers published from 2006–2015 has steadily increased across the years, ranging from 12 publications in 2006 up to 110 in 2015. On average, the number of publications has increased by +32.37%, with two peak points: one between 2009–2010 (+55%) and between 2012–2013 (+128%; Figure 1).

### 3.4 Publications in journals with and without impact factor

To highlight the quality of the scientific output of nursing research in Italy, papers were ranked according to the journal name and their impact factor (Table 2). Of the top 29 journals, 15 had an IF ranging between 0.236–3.755. The impact factors are updated to 2016.

**Table 2:** List of the top 29 journals where the papers from our PhD Schools of Nursing have been mostly published between 2006–2015

Ranking	Journal name	Papers (N)	Impact factor
1	Professioni Infermieristiche	42	–
2	Infermiere Oggi	27	–
3	L'Infermiere	22	–
4	Scenario	19	–
5	Journal of Advanced Nursing	16	1.998
6	Annali di Igiene	13	–
7	Assistenza Infermieristica e Ricerca	13	0.236
8	Nursing Ethics	12	1.755
9	European Journal of Cardiovascular Nursing	11	2.763
10	International Nursing Perspectives	10	–
11	Igiene e Sanità Pubblica	7	–
	Nurse Education Today	7	2.533
12	Giornale Italiano di Scienze Infermieristiche Pediatriche	6	–
	Journal of Clinical Nursing	6	1.214
	Medic	6	–
	Health Professionals Magazine	6	–
13	Professione infermiere Umbria	5	–
	Medicina del Lavoro	5	
	European Journal of Oncology Nursing	5	1.826
	International Journal of Nursing Studies	5	3.755
14	Annali dell'Istituto Superiore di Sanità	4	0.899
	Children's nurses ‐ the Italian Journal of Pediatric Nursing	4	–
	Nurse Education in Practice	4	1.314
	International Nursing Review	4	1.517
	Journal of Nursing Scholarship	4	2.396
	Pain Nursing Magazine	4	–
	BMC Medical Education	4	1.572
	International Emergency Nursing	4	1.298
	Italian Journal of Nursing	4	–
	Supportive Care in Cancer	4	2.698

**Figure 2:**
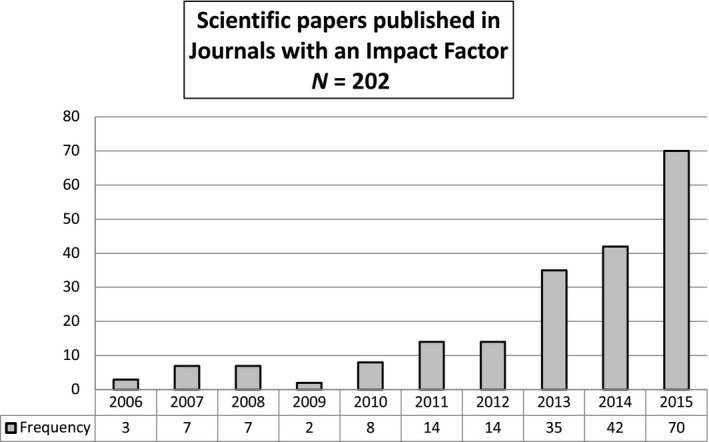
Scientific papers published in Journals with an Impact Factor

We analysed how many papers were published in journals with an impact factor across the 10‐year period of our study and found that the total number of publications has steadily increased over the years, especially between 2011–2015 (Figure 2). Until 2010, the mean number of papers published in journals with an impact factor was 5.4 a year. Starting from 2011, we observe a first rapid increase with 14 publications, reaching a peak of 70 papers in 2015.

**Figure 3:**
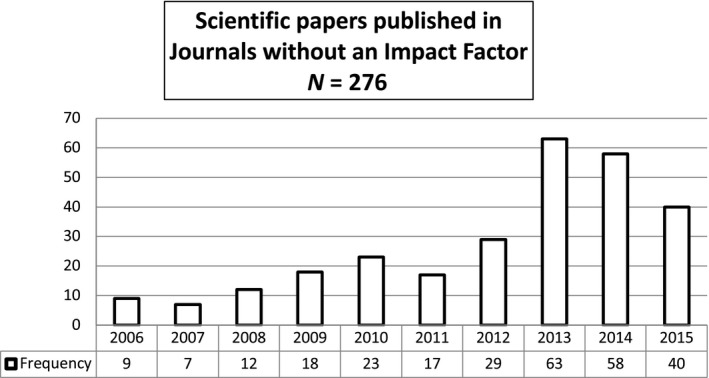
Scientific papers published in Journals without an Impact Factor

With regard to the number of papers annually published in journals without an impact factor, we observed a non‐linear trend across the 10‐year period (Figure 3). The number of papers in journals without an impact factor increased between 2006 (*N* = 9)–2010 **(** *N* = 23**)**; then, this number dropped by about 26% in 2011, but then increased rapidly again with a peak of 63 papers in 2013. Between 2014–2015, this number gradually dropped to 40 papers in 2015.

### 3.5 Publication topics

**Figure 4:**
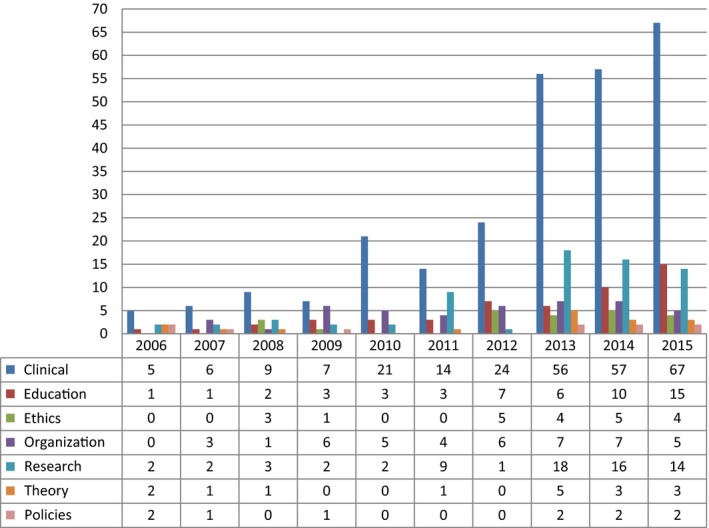
Number of papers published on each topic and their distribution from 2006–2015

Most of the publications of the PhD Schools of Nursing mainly focused on clinical and healthcare aspects (55.64%; *N* = 266), followed by research methods (14.44%; *N* = 69), education (10.67%; *N* = 51), organization/management (9.2%; *N* = 44), ethics (4.6%; *N* = 22), theories (3.35%; *N* = 16) and policies (2.1%; *N* = 10; Table 3, Figure 4). Across the 10‐year period, the number of papers focusing on clinical aspects has always been significantly higher than all the other topics. Of the papers on other topics, in 2015 those on nursing education (*N* = 15) outnumbered for the first time those on research topics (*N* = 14).

**Table 3:** Number of papers published on each topic from 2006–2015

	Clinical	Education	Ethics	Organization	Research	Theory	Policies
Total papers	266	51	22	44	69	16	10
%	55.64	10.67	4.6	9.2	14.44	3.35	2.1

### 3.6 Topics in journals with impact factors

Between 2006–2015, 202 scientific articles were published in journals with an impact factor and 276 in journals without an impact factor. The impact factors of the journals where the papers were published ranged between 0.18–19.896. Also, in this case, 60.8% (*N* = 152) of the papers published in journals with an impact factor were on clinical topics, and of these, 43.4% (*N* = 66) were published in journals with an impact factor between 2.0–20.0. These were followed by papers on research topics (*N* = 41; 16.4%) and education (*N* = 21; 8.4%). Interestingly, of the 13 papers on ethics, 12 were published in journals with impact factors that ranged between 1.5–1.99 (Table 4).

**Table 4:** Impact factors distributed according to the topics of the papers

Topics	IF 0–0.99	IF 1.0–1.49	IF 1.5–1.99	IF 2.0–20.00	Total	%
Clinical	27	20	39	66	152	60.8
Education	4	5	7	5	21	8.4
Ethics	1	0	12	0	13	5.2
Organization	5	1	3	1	10	4.0
Research	11	7	12	11	41	16.4
Theory	0	1	1	1	3	1.2
Policies	5	1	3	1	10	4.0

## 4 DISCUSSION

Since 2006, when the nursing doctoral schools were founded in Italy, the number of articles published in scientific journals has increased in a yearly basis from 12–110, especially from 2010, when the first doctoral students completed their programme. The small number of students admitted to the nursing doctoral schools in the first years has inevitably limited the number of scientific publications. However, proportionally to the number of doctoral students (*N* = 79), the number of papers published has been high (*N* = 478), with an mean of six papers per person. Over time, the doctoral schools have gradually developed their own research lines by focusing on more specific areas and topics. In addition, the quality of the scientific papers has increased, and this was demonstrated by the fact that in the last few years most of the papers are published in scientific journals with higher impact factors, especially starting from 2013 and on clinical topics. This has happened despite the application of nursing research findings to clinical practice is still difficult or slowed down by the limited accessibility to scientific journals and by the language barrier, since most are published in English (Oermann et al., 2008).

Another aspect that emerged from this survey was that in the last few years, nursing studies have started to be conducted on a much wider scale despite limited funding and resources. An example of this is provided by the RN4CAST@IT study, which involved 42 hospitals across the whole country (Sasso et al., 2016) showing clinical nurses how research can bring about change to nursing practice. Italian nursing doctoral schools have also accelerated the validation of many assessment tools, which have an important impact on the quality of care and over 70% of the publications of the four doctoral schools focused on the scope of clinical practice, with a substantially even distribution across the four schools. Approximately 11% of the publications focused on research topics, 4.75% focused on education, 0.5% on theory, 7.75% on organization, 5% on ethics and only one paper on political aspects. Therefore, in Italy very little nursing research has been conducted in the fields of ethics, theory, organization and politics. This trend was also described in a study conducted by Zanotti and Pecile in (2002)—before the doctoral schools of nursing were established—which showed that out of 300 papers published between 1983–2001, most focused on clinical practice, using principally observational methods, highlighting the need for advanced education in the field of research to value the role of nursing. Since, 2006, nursing doctoral schools have steadily improve the scientific and methodological rigour of nursing studies, confirmed by the increasing number of papers published in journals with higher impact factors.

Also, findings related to the topics of scientific papers are consistent with those of similar studies conducted in other countries. For instance, in Europe a significant contribution was provided by the Irish nursing literature. After analysing all the research papers published until 2005, the fields of publication in Ireland were clinical practice (56%), nurses’ professional education (25%) and nursing management or professional issues (19%) (McCarthy, Hegarty, & O'Sullivan, 2006). These data show how through the analysis of the literature produced in a specific sector it is possible to obtain an outline of its scientific and cultural evolution. In some countries instead, these data are partly discordant. For instance, in Brazil in 2006, most nursing publications focused on education (19%), followed by community health (11%) and adult health (11%) (Silva, Oliveira, Frota, & Fialho, 2008).

Polit and Beck (2009) found significant differences in the types of nursing topics mostly published across the regions of the world. In Europe, Canada and Australia nursing research studies mainly tended to focus nurses themselves, whereas in Asia and the United States the focus was mainly on patients. Polit and Beck (2009) also found that in Europe, countries like the United Kingdom, Norway and Sweden nursing research mainly adopted qualitative methods. This interesting finding was also confirmed by Meleis (2011), who reported that in the field of research, at the beginning professionals tend to study themselves as a profession and their own preparation and organization; then, they shift their attention towards patient care and the definition of their own care models.

The analysis conducted on the publication activities of the doctoral schools highlights how the production of nursing knowledge reflects Italy's historical–cultural fabric and the evolution of the doctoral schools from when they were established to today, through the fields of research it has focused on. Our findings align with the statement of the NINR, that the main purpose of nursing research should be to study through clinical research those phenomena that are associated with the provision of direct care to the person.

A study that analysed scientific publications before doctoral schools of nursing were opened in Italy highlighted the necessity to explore and investigate the professional value of nursing and consequently improve the quality of care (Sansoni, Comerci, & Marucci, 2006). This need started to be addressed through the opening of the doctoral schools of nursing, which have increased nurses’ knowledge in terms of research methodology and scientific rigour, by conducting scientific studies both in Italy and internationally, contributing to the assertiveness of the nursing profession. Moreover, doctoral schools in Italy have increased nurses’ general knowledge, offering greater potentials in terms of resources and competencies. This has also facilitated the creation of a new generation of nursing leaders, who can offer a new vision based on scientific evidence and capable of interacting with institutions and participate in decision‐making with other professionals to resolve healthcare issues.

### 4.1 Limitations

Since, it was not possible to have the full mailing list of all the students who had completed the doctoral programme from the various doctoral schools, we were unable to conduct a precise calculation of the response rate. However, after checking Web of Science, we subsequently found that the response rate was very good.

Another limit is that we did not include papers of those who did not accept to participate. Finally, although in Italy there is very little funding for nursing research, an interesting aspect we did not analyse was how many studies received funding and by whom.

## 5 CONCLUSIONS

In the last 10 years, nursing doctoral schools have greatly contributed to the development of nursing research, not only in terms of academic excellence, but also in producing a wealth of scientific evidence that is relevant and applicable to Italian clinical settings. However, academic nurse leaders need to identify strategies to obtain more funds for nursing research projects. For instance, universities could form partnerships with healthcare institutions to conduct research and support them to design and develop studies to meet the demands of clinical services, patient outcomes and the quality and safety of care. In these cases, grant applications for projects with a clinical focus are instrumental (Wilkes, Cummings, Ratanapongleka, & Carter, 2015).

However, the availability of funding and resources for nursing research in Italy are still scarce. This is mainly due to the persistence of a culture that gives little priority to nursing research and this constitutes a barrier to the further development of nursing studies. This requires doctoral schools to develop more studies that focus on policies and on the financial impact and economic value of nursing, which until now have been very few (only 10). In this way, nursing research projects could have more chances of attracting funding from Italian governmental and health institutions. In other countries, such as the USA and several North European countries, the role of doctoral schools is greatly valued and many institutions and agencies fund nursing research projects that produce better patient outcomes and constantly improve the quality of health care. Many studies are also used as benchmarks and provide indicators that enable to improve healthcare standards and are instrumental to identify and develop new lines of future research. Italian nursing doctoral schools now have the potential to become an integral part of a national funding system for the improvement of health care and boosting a widespread recognition of the inestimable value of their work.

## CONFLICT OF INTEREST

None.

## AUTHOR CONTRIBUTIONS

AB, RW, LS: Study design. MB, RP, VB, LC, ND, DV: Data collection**. **GC, MZ, ND, MB, RP: Data analysis. RW, AB, LS, FT, GA: Study supervision. RP, MB, GA, FT: Manuscript writing. RW, AB, LS, AB, GA, FT: Critical revisions for important intellectual content.

## PATIENT CONSENT STATEMENT

No patient consent was required for this study.
